# Leadership Identity Development: Insights Into the Lived Experiences of Undergraduate Business Students on a Placement Year

**DOI:** 10.1002/yd.70052

**Published:** 2026-05-15

**Authors:** Graziana Di Pede, Chris Turner

**Affiliations:** ^1^ University of Sussex Business School Brighton UK; ^2^ University of Reading Reading UK

## Abstract

The proliferation of leadership courses offered by UK business schools reflects a growing emphasis on leadership development, potentially driven by an increased demand for leaders in the labor market alongside a desire to prepare students for future leadership roles. This study explored how pre‐ and early‐career experiences affect undergraduate business students’ leadership identity development over time. Results showed that their development was strongly influenced by observed role models as well as enacted roles and responsibilities within specific contexts. Implications for both theory and practice are discussed.

## 1 Introduction

Business schools view students’ leadership development as an important objective (Zaar et al. 2020). To achieve this, they should be providing students with more opportunities to gain practical experience. A study conducted by the Chartered Management Institute (CMI 2018) showed that business and management students strongly aspire to become leaders and increasingly value work experience to enhance their employability and chances to get into leadership positions later in their career. This is also in line with literature that advocates for more practice‐based learning in the field of leadership development (e.g., Preston and Floyd 2016).

Experiences like work placements undertaken as part of their studies could play a significant role in leadership development. Accordingly, the aim of this study is to explore the impact of these early‐career experiences whilst also considering previous experience in other contexts such as family life, school, and university, referred to as a pre‐career stage.

Most research so far has focused on the leadership development of people who are already in managerial roles (e.g., Kempster 2006), neglecting the development of business and management students, especially in a work‐based context. Furthermore, there are aspects of learning from experience that are still under‐explored, for example, observational learning (Kempster and Parry 2014).

This study addresses this gap by examining observational as well as experiential learning in both pre‐ and early‐career experiences although the main focus is on the latter. Hence, the guiding research question in this study is *“How do pre‐ and early‐career experiences affect the development of undergraduate business students’ leadership identity?”*. This can be seen as a gradual process as students gain experience over a prolonged period and could engage in deeper reflection to interpret their lived experience.

## 2 Leadership Learning as Identity Development

Extensive research has shown that leadership learning and identity development are inextricably intertwined (Di Pede 2024; Kempster and Stewart 2010; Komives et al. 2006; Zaar et al. 2020). Accordingly, it can be argued that through leadership experience in various contexts such as academia and the workplace (Di Pede 2024; Hammond et al. 2017), individuals can begin to form and develop a leadership identity, which can be described as “multiple, temporary, changing and socially constructed” (Schedlitzki and Edwards 2018, p. 301).

Arguably, experiences such as early‐career events, major tasks and projects, and working alongside effective (and ineffective) leaders are crucial to leadership development (Longenecker and Insch 2019). Through reflection on these experiences, knowledge of leadership is acquired, usually in a tacit subconscious fashion, and then applied to future leadership roles and activities (Kempster 2006, 2009). This process of becoming a leader (Kempster 2006) suggests identity is the product of both observation and experimentation followed by a period of critical evaluation (Ibarra 1999), which may be enhanced through discussions with more experienced colleagues.

In line with Bandura's (1986) social learning theory, it can be argued that observing positive and negative role models (Gibson 2003, 2004) in different contexts affects individuals’ understanding of effective and ineffective leadership, and consequently their actions as they seek to emulate behaviors that result in desired outcomes and avoid those that would result in undesired ones (Di Pede 2024). Similarly, working alongside significant others over time, may lead to the acquisition of a generic understanding of leadership, which is then refined and affirmed in later career stages (Kempster and Parry 2014; Mumford et al. 2000). Furthermore, exposure to female role models is likely to have a positive impact on women's leadership identity, especially when they can identify with these role models and regard their success as attainable (Hoyt and Simon 2011). Accordingly, having more female leaders at all levels of an organization will greatly benefit women's leadership identity development (Ibarra et al. 2013; Murphy et al. 2017).

However, leadership learning through lived experience often happens in a sub‐conscious manner (Di Pede 2024; Kempster 2006), and thus, reflective engagement and interpretation are necessary to make sense of these accumulated experiences (Devies 2022; Hibbert et al. 2017). By extracting insights from specific events, also known as ‘critical incidents’ (Cunningham 2008), and by engaging in meaningful dialogue with others, leaders deepen their knowledge and understanding of effective leadership (Hibbert et al. 2017).

This sense‐making process produces leader competence (Hammond et al. 2017). More specifically, it may result in ‘leader development’ through the acquisition of individual knowledge, skills, and abilities (KSAs) associated with formal leadership roles and ‘leadership development’ through collaboration with others (Day 2000). Furthermore, research suggests that people that strongly identify as a leader, are more likely to seek out opportunities to practice leadership, and thus, enhance their existing KSAs as well as acquire new ones (Day and Sin 2011; Hammond et al. 2017). The competence developed over time allows expert leaders to tackle increasingly more complex problems by relying less on memory and more on intuition (Lord and Hall 2005).

Finally, leader identity can range from *fully integrated* if someone perceives himself/herself as a leader in all areas of his/her life to *integrated across some domains*, for example, work and community but not necessarily at home, and *splintered* or domain specific, for example, only at work (Hammond et al. 2017). Arguably, by integrating leadership knowledge with their identity, individuals develop an “expert and unique manner of leading” (Lord and Hall 2005, p. 611).

## 3 Leadership Identity Development in University Students

The development of leadership identity amongst university students has gained increasingly more attention in recent years, with scholars emphasizing both theoretical models and the role of experiential learning. Central to this discourse is the Leadership Identity Development (LID) model proposed by Komives et al. (2006), which outlined a six‐stage progression from Awareness (stage 1), where students seem to recognize leadership in others (e.g., parents and teachers), but not necessarily in themselves, to Integration (stage 6) where they begin to see themselves as leaders through involvement in group activities and formal roles.

Di Pede's (2024) research extended these insights to professional contexts by exploring how undergraduate business students learn to lead in early‐career experiences. Her findings closely aligned with the LID model, demonstrating how students progress through similar developmental stages as they engaged with real‐world challenges, observed role models, and took on increasing responsibility during their work placement.

Complementing these findings, Zaar et al. (2020) conducted a large‐scale qualitative study with 510 business students from a Northern‐European university, categorizing them into four identity levels: *weak*, *provisional*, *moderate*, and *strong*. Students with weaker identities often lacked leadership experience and held fixed views of leadership as innate, demonstrating a low level of development (Hammond et al. 2017). Conversely, those with stronger identities reported substantial prior experience and a belief in leadership as a learnable skill. This is consistent with a previous study conducted by Day and Sin (2011), which found an ongoing association between leadership identity and leadership effectiveness. Despite the much smaller sample of 13 students, Di Pede's (2024) work corroborated Zaar et al.'s (2020) study results by showing that most students either already viewed themselves as leaders or wished to become one in their future career after gaining more work experience. This supports the notion that accumulated experience is crucial to leadership identity development.

However, the process of developing leadership identity is not without its difficulties. Scholars such as DeRue and Wellman (2009), Benjamin and O'Reilly (2011), and Longenecker and Insch (2019) highlighted the importance, and potential pitfalls, of developmental challenge. Whilst exposure to difficult experiences can act as an accelerator of leadership learning, excessively demanding experiences may have the opposite effect by overwhelming students and stifling reflection. This complexity was evident in Di Pede's (2024) study undertaken during the Covid‐19 pandemic. In fact, although many students regarded this crisis as a valuable leadership learning experience, some felt intimidated by the increased responsibility that leaders bore during that time, which dampened their enthusiasm for future leadership roles. This illustrates the double‐edged nature of challenge in leadership development: beneficial when balanced, but detrimental when excessive.

## 4 Research Design and Methodology

The primary purpose of this research study was to gain a deeper understanding of how pre‐ and early‐career experiences affected the development of students’ leadership identity, especially within a work‐based context. To understand these experiences from the students’ perspective, narrative inquiry was chosen as the main research strategy. Through storytelling, participants provide an interpretation of specific events whilst the researcher analyses the meanings that the participants attach to these events (Saunders et al. 2016).

Narratives about leadership experiences were collected through semi‐structured interviews and reflective journals. Each student was interviewed twice; before and after the placement. The interviews conducted prior to the placement were used to explore students’ understanding of leadership in the pre‐career stage. Conversely, the interviews undertaken at the end of the placement year were designed to investigate the acquisition of contextual leadership knowledge in the early‐career stage. Before the post‐placement interview, students were required to draw a timeline of events to identify critical incidents and people that may have influenced their understanding of leadership throughout the placement year. This exercise proved to be particularly effective in extracting participants’ tacit knowledge of leadership as demonstrated by Kempster (2009).

Whilst on placement, students were also asked to write two reflective journals to record their thoughts, and observations regarding two leadership events witnessed in the workplace. Data were collected before, during, and after the work placement over a whole academic year. This longitudinal design was crucial to study this phenomenon as leadership identity development is a dynamic and longitudinal process in itself.

### 4.1 Sample Selection

The study involved nine female and four male placement students from one business school based in the South of England and were all aged between 18 and 21 years old. In line with purposive sampling, participants were chosen on the basis of specific characteristics (Cohen et al. 2017). In this case, the selection requirements were: (a) generic understanding of leadership acquired through their studies, and (b) opportunity to gain contextual leadership knowledge during their work placement.

All 13 students were informed and invited to participate via e‐mail and by word of mouth. Nine students volunteered from the first cohort, which comprised a total of 91 placement students in the academic year 2019–2020. Four more students were recruited from a second cohort consisting of 94 placement students in the following academic year (2020–2021) for a total of 13 participants. The main characteristics of the sample are summarized in Table 1.

**TABLE 1:** Details of research participants (names changed to protect anonymity).

Participant	Gender	Bachelor's degree	Placement cohort
Frankie	Female	Management	2019–2020
Jessy	Female	Management	2019–2020
Vivian	Female	Accounting & Finance	2019–2020
Laura	Female	Accounting & Finance	2019–2020
Sarah	Female	Accounting & Finance	2019–2020
Katie	Female	Accounting & Finance	2019–2020
Monica	Female	Management	2019–2020
Richard	Male	Strategy and Marketing	2019–2020
Mary	Female	Management	2019–2020
Helen	Female	Management	2020–2021
Jamie	Male	Management	2020–2021
Tom	Male	Accounting & Finance	2020–2021
Mark	Male	Accounting & Finance	2020–2021

Most pre‐placement interviews took place between May and September 2019. Some of them were face‐to‐face and others were on Skype. They lasted approximately 20 minutes and were all recorded and transcribed. Post‐placement interviews with the first cohort were carried out via Skype between July and September 2020 and lasted about 30 minutes. Between the two interviews, students were asked to submit two reflective journals, one in November 2019 and the other in March 2020. Interviews and reflective journals with the second cohort (2020–2021) followed a similar timeline. Overall, 26 interviews were undertaken in addition to the pilot interviews, and 24 reflective journals were collected and analyzed.

### 4.2 Data Analysis

The approach taken in this study was *thematic narrative* analysis, which according to Shukla et al. (2014) can be seen as two distinct (i.e., thematic and narrative) but complementary analytic methods that when combined, allow for a deeper understanding of qualitative data. Thematic analysis was used to identify patterns and variations across cases and to develop an overall understanding of the dataset. Narrative analysis, by contrast, allowed closer attention to individual experiences and their distinctive features.

After searching for specific theory‐driven themes across all of the narratives, each narrative was analyzed in more depth through a story map adapted from Richmond (2002). According to this, the students’ leadership experiences were categorized into past, present and future intentions and analyzed in relation to self, family life, education and workplace.

Consistent with narrative inquiry, the research process was understood as interpretive and relational. Iterative analysis was used to remain attentive to how the researcher's role and assumptions may have shaped data interpretation while foregrounding participants’ meanings.

## 5 Results

**FIGURE 1:**
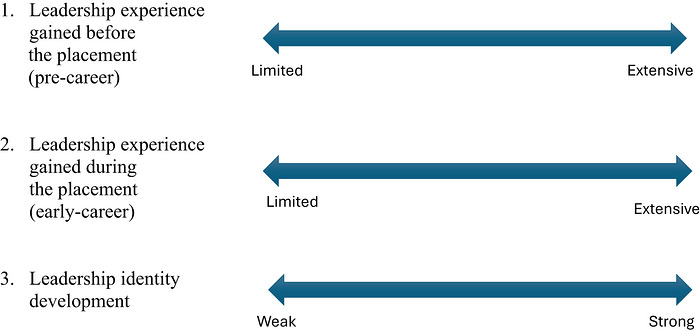
Dimensions of leadership experience and identity development.

Each of the students included in the research sample could be located according to the following dimensions (Figure 1):

Each of these dimensions represented a continuum along which individual students could be placed (Table 2).

**TABLE 2:** Continuum of responses from individual students.

	Experience before the placement			Experience during the placement		
	Limited	Medium	Extensive	Limited	Medium	Extensive
Experiences of leadership before and during the placement	**Katie**	**Laura** Frankie Monica Mary Jamie Sarah Tom Mark Richard	**Vivian** Jessy Helen		Jessy Sarah Tom Richard	**Vivian** **Katie** **Laura** Frankie Monica Mary Helen Jamie Mark

	**Leadership identity**					
	**Before the placement**			**After the placement**		
	Weak	Moderate	Strong	Weak	Moderate	Strong
The development of a leadership identity	**Katie** **Laura** Sarah	Frankie Monica Mary Helen Jamie Tom Mark Richard	**Vivian** Jessy	Sarah	**Laura** Mary Tom Mark Richard	**Vivian** **Katie** Jessy Frankie Monica Helen Jamie

The first dimension refers to how much leadership experience the students had before their placement. The second dimension represents the students’ leadership experience during the placement. Finally, the third dimension refers to the students’ leadership identity before and after the placement, which ranged from relatively weak to strong.

This section will now discuss the findings in more depth by looking at three different types of students that emerged from the sample. First, the ‘confident’ student, that is, participants that demonstrated a strong leadership identity before and after the placement, possibly due to their extensive leadership experience from a relatively young age (e.g., Vivian). Second, the ‘unconfident’ student, that is, participants that did not identify themselves as a leader before their work placement but displayed a much stronger leadership identity after it (e.g., Katie). Third, the ‘cautious’ student, that is, participants that displayed a weak to moderate leadership identity throughout the placement, (e.g., Laura).

### 5.1 The Confident Student

Vivian was a second‐year Accounting and Finance student when she started her placement as a Tax Corporate Assistant in an accounting firm. Interview data revealed that Vivian already had a strong leadership identity prior to her work placement. In fact, she described herself as follows:

“I'd probably say that I'm a *natural* leader. […] I think because I'm confident everyone sorts of just assumes that I'd be happy to lead a group, and I am. It's not an issue but I think it comes *naturally* to me because I'm not bothered by what anyone thinks of me. If I ask you to do something and you don't want to do it, you just have to do it”.

(Vivian, pre‐placement interview)

This suggested Vivian strongly identified as a leader and that she was confident in herself and her leadership skills (e.g., being assertive). Whilst the notion of ‘natural leader’ should be challenged as it precludes the idea that such skills can be nurtured and developed, other students used similar words when discussing their own leadership identity. For example, Jessy argued that leadership was “*embedded into her*”, that it was a role that she had “*always naturally taken*”, and that she had “*a natural sense to lead people”*.

From a leadership identity perspective, such claims can be understood as the product of early leadership experiences and a strong sense of self‐efficacy, rather than inherent ability. Framing leadership as “natural” risks reinforcing traditional and fixed beliefs about who can lead and downplay the developmental and relational nature of leadership learning.

**FIGURE 2:**
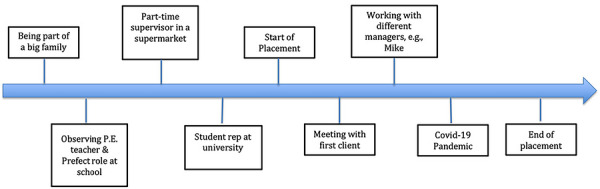
Overview of Vivian's experiences relevant to leadership and identity development. N.B. The line represents time encompassing home and family environment, school, university, and work before and during the placement. This timeline is not to scale and only serves to act as an illustration of events over time. The same applies to Figures 3 and 4.

Interestingly, Day and Sin (2011) found people who hold a strong leadership identity are more likely to seek opportunities to exercise it. This may then play a significant part in their leadership identity development. This is certainly the case for Vivian, who had a considerable amount of leadership experience throughout her life as illustrated in Figure 2.

Before the placement, Vivian had already experienced leadership in various contexts including family life, school, university, and the workplace. For example, in the pre‐placement interview, she explained how observing positive role models such as family members and her P.E. teacher at school had influenced her understanding of effective leadership, which she regarded as *“someone that guides and helps people”*.

Furthermore, in line with stage 2 (exploration) of Komives et al. (2006) leadership identity model (LID), Vivian had sought opportunities to lead from a relatively young age as shown in the following interview extract:

“Since I was a child, I've always felt like I've been in charge. When I was younger and then through school, I was a Prefect in Year 11, a senior Prefect in year 13. […] I'd say specifically at work, there are two supervisors and then there's me, who's like a part‐time supervisor […]. Last year and this year, I've been Student Rep for Accounting and Finance”.

(Vivian, pre‐placement interview)

Similarly, during the placement, Vivian experienced leadership mainly by taking on responsibility for some challenging tasks as well as by working with more senior colleagues. One event that seemed to be significant to her was meeting her first client as illustrated below:

“Another event was when I went to visit my first client with Mike. He made sure I was comfortable talking to the client. He let me take some responsibility for the work. He oversaw my communications with the client and helped me plan what I was going to ask and how to use that information”.

Reflecting on this event, she then added:

“It was a bit scary… I was scared at first. But when I went in there, I was introduced to the client and the rest of their team, which I think put me at ease a bit more”.

(Vivian, post‐placement interview)

This event can be seen as a critical incident in Vivian's professional life as it was something that she had not done before, and thus, required her to assume new responsibility (Cunningham 2008). Whilst it appeared to have a negative connotation at first (scary), it was clearly seen as a significant experience in the longer term as Vivian identified the event as a crucial leadership learning experience (see Figure 2). Arguably, Vivian was able to recall this specific episode due to its emotional significance as well as the involvement of another person, that is, her supervisor Mike, as suggested by Kempster (2009).

By working with Mike, Vivian gained a deeper understanding of the kind of leader that she aspired to become:

“Supportive, encouraging, happy to answer questions, empathetic, someone who doesn't just understand how you feel, but they can put themselves in your shoes”.

(Vivian, post‐placement interview)

This corroborates the ideas of Gibson (2003, 2004), who suggested observing role models in the workplace affects individuals’ professional identity as they redefine the latter in relation to others (Lord and Hall 2005).

The evidence presented here suggests that Vivian developed a strong leadership identity by accumulating leadership experience in multiple domains. She demonstrated considerable awareness of her own leadership qualities (e.g., assertiveness) as well as a willingness to take on leadership roles and responsibilities from a relatively young age.

This is in line with modern research that shows that leadership is primarily learned rather than born. It is also consistent with Zaar et al.'s (2020) study, which found that individuals with a strong leadership identity believe that they already have enough leadership experience as well as key leadership qualities. Nonetheless, this might mean that the individuals concerned may develop a tendency to be over‐confident in their own abilities. Vivian's leadership identity also appeared to be fully integrated as she perceived herself as a leader in all areas of her life including education and the workplace (Hammond et al. 2017).

### 5.2 The Unconfident Student

**FIGURE 3:**
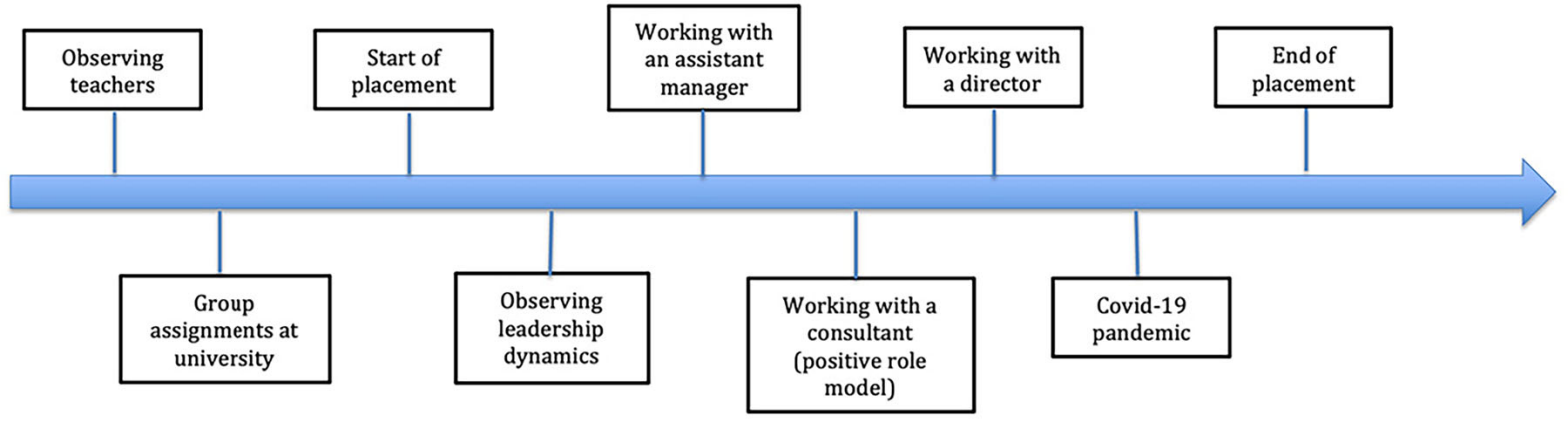
Overview of Katie's experiences relevant to leadership and identity development.

Like Vivian, Katie had also just finished her second year of university when she started her work placement in an accounting firm based in the South of England. Throughout the placement year, Katie undertook a remarkable personal transformation from shy young woman with no confidence in her leadership abilities to a much more confident person aspiring to more senior leadership positions. This is a story worth telling as it demonstrates the power of early‐career experiences in shaping identity and aspirations. Figure 3 is an overview of Katie's leadership journey.

It is worth noticing that compared to Vivian, Katie had little leadership experience before starting her work placement. However, like Vivian, she had the opportunity to work alongside several significant others (e.g., managers and directors) during it.

Pre‐placement interview data suggested that prior to the placement, Katie had experienced leadership only within an educational context, for example, group assignments. When asked about whether she had found herself in situations where she had to exercise leadership, she commented:

“Not often because I'm quite shy, which is something I'd like to work on. From my placement I'd like to become more confident. In the first term of the first year, we had to do a project where we were in teams. So, I sort of took a bit of leadership to tell people what they can do to get it done”.

Reflecting on how challenging this was for her, she then added:

“A little bit (challenging) because I don't like telling people what to do. Especially when you can tell they don't want to do it”.

(Katie, pre‐placement interview)

In stark contrast with Vivian, who identified as a confident and assertive leader, Katie described herself as shy and unconfident, and thus, not fully able to lead others. In fact, unlike Vivian, Katie struggled with giving others explicit orders. This is something that she was clearly determined to change by gaining more self‐confidence through work experience.

It is also worth noting how Katie may have not proactively sought opportunities to exercise leadership due to her lack of self‐belief. This further corroborates the ideas of Day and Sin (2011), who argued that people are more likely to develop as leaders if they identify as one. Accordingly, Katie's leadership development may have been initially hindered by self‐doubt.

During the placement, Katie worked alongside more experienced colleagues as well as observed interesting leadership dynamics. One experience that seemed particularly crucial to Katie's leadership learning was working with a consultant, for whom she expressed strong admiration in both the post‐placement interview and in one of her reflective journals. In the latter, she reflected on her experience of helping the consultant during a particularly busy time at work as reported below:

“I was made to feel as if the work I did often contained minimal errors and confidence was placed within my work, which developed my confidence and ultimately motivated me to complete this work”.

(Katie, reflective journal)

In this case, the consultant's feedback and support boosted Katie's motivation and confidence to complete a challenging task as shown in the following journal extract:

“The consultant was communicating instructions clearly and concisely, whilst being open for questions to ensure I understood how to properly complete the task at hand. Her leadership became apparent when she encouraged me to continue when I felt like I wanted to give up”.

(Katie, reflective journal)

Furthermore, Katie reflected on the importance of delegation:

“Distributing work that is the happy medium between simple and challenging is what makes a leader a leader. […] My consultant was able to identify that I could complete this task despite the fact that I would find it challenging.”.

(Katie, reflective journal)

In other words, Katie praised the consultant's ability to delegate a task that was neither too simple nor too difficult whilst firmly believing that she could complete it. This finding is consistent with that of DeRue and Wellman (2009) who argued that a developmental challenge is beneficial only up to a certain point as experiences that are perceived as too challenging can overwhelm individuals and consequently hinder their learning.

Working alongside this consultant proved to be an invaluable developmental opportunity for Katie. She not only enhanced her understanding of effective leadership, (e.g., supporting and trusting others), she also realized that one day she would be in the position to lead less experienced colleagues (novices) herself. In particular, she learned that:

“Leadership skills are quite likely to be learned by watching those around me and those who teach you. I feel I have learned an effective way to help someone less experienced than myself to complete similar tasks”.

(Katie, reflective journal)

This experience illustrated Komives et al. (2006) idea of transition from viewing only other people as leaders (e.g., the consultant) to starting to perceive oneself as a leader. In fact, whilst Katie displayed a rather weak leadership identity at the start of her placement mainly because of her personality (shy and unconfident) as well as lack of experience, this certainly changed throughout the placement as she became more knowledgeable and confident as illustrated in the following comment:

“My confidence has increased so much. I didn't realise how much of a difference a placement would make to my confidence”.

(Katie, post‐placement interview)

When asked whether she aspired to a leadership role in her future career, she commented:

“Yes, I think I do now. I think before my placement, I would have said no. I would have been too afraid that I couldn't necessarily handle being a leader for other people. I think now, because of my placement, I think I could take on a leadership role, whatever that might be. So, once I go back, I would be at consultant levels. I also think that one day I would definitely aspire to be a director, which obviously would have high leadership roles within the company”.

(Katie, post‐placement interview)

This shows the crucial role that early‐career experiences play in leadership identity development (Di Pede 2024). During the placement, Katie's leadership identity developed from weak to strong (Zaar et al. 2020) as she began to envision herself in senior leadership roles at least in one domain, that is, the workplace (Hammond et al. 2017). Other students had a similar experience. For example, Jamie stated that before the placement he could not really see himself pursuing senior leadership positions whereas after the placement he wished to *“improve his leadership skills and go up the leadership ladder”*.

As for the kind of leader Katie wished to become, she claimed:

“A leader that takes a lot of the values that the consultant I talked about before had”, which included “positive, welcoming, and good at explaining things”.

(Katie, post‐placement interview)

It can be inferred that Katie's leadership identity and aspirations were strongly influenced by this colleague, who can be seen as a positive female role model (Gibson 2004; Hoyt and Simon 2011) and a key developmental influence (Komives et al. 2006).

Like Katie, other female students discussed their experience of observing women leaders during their work placement, which demonstrates the importance of female role models to identity development (Ibarra et al. 2013).

### 5.3 The Cautious Student

**FIGURE 4:**
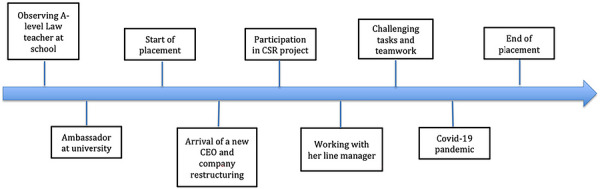
Overview of Laura's experiences relevant to leadership and identity development.

Laura was a second‐year Accounting and Finance student when she began her work placement in a travel company based in the South of England. Laura did not identify as a leader prior to the placement, and she remained hesitant toward seeking leadership roles even after the placement. Figure 4 provides an overview of Laura's leadership experiences before and during the placement.

Before the placement, Laura experienced leadership mainly within an educational context as she discussed her A‐level Law teacher as a positive role model, and her role as an Ambassador for the Widening Participation team at university. During the placement, Laura witnessed major changes, such as the arrival of a new CEO, and how the company's leaders dealt with the challenges brought on by the Covid‐19 pandemic. The latter appeared to have had a major impact on Laura's leadership learning. This event fits Cunningham's (2008) definition of a critical incident as it created “a significant disturbance” in Laura's professional life and of “her understanding of effective practice” (p.166) as shown in the following excerpt:

“As a travel company that works on a volume basis the current crisis which has led to the grounding of flights and shutting of borders globally has had a profound impact on the company. Due to this and not knowing how long it would last for, the company had no choice but to make some very difficult decisions to essentially try and ensure the company would survive the coming months with little/no revenue”.

(Laura, reflective journal)

The Covid‐19 crisis provided Laura with the opportunity to gain an understanding of how leaders deal with unprecedented challenges and hardships, which can be seen as useful experiences for leadership development (McCall 2010).

Looking back on this experience, Laura praised the company's leaders for how they handled the crisis as reported in her journal:

“The Senior Leadership team have been professional, clear and realistic. They haven't tried to sugar‐coat anything but at the same time they have voiced any positive messages where possible. I'm very confident that the behaviours used were appropriate as they communicated the seriousness of the situation to ensure everybody understood why they were putting certain things in place but equally they made you feel assured that they weren't taking the actions lightly and were clearly researching the options open to them very thoroughly”.

(Laura, reflective journal)

Accordingly, Laura emphasized the importance of effective communication and maintaining a positive mindset during adversity. This event not only influenced Laura's understanding of effective leadership, but also her definition of leadership, which she described as *“the bearing of responsibility for a group”*. Undoubtedly, the leaders of the company that she worked for bore a great amount of responsibility having to make some difficult decisions because of the pandemic. Witnessing such hurdles had a somewhat negative impact on Laura's leadership identity.

Like Katie, Laura did not identify as a leader before the placement as shown in the following extracts:

“At the moment, I don't (aspire to become a leader) because I think that's such a massive jump away, but I definitely wouldn't not consider it, if that makes sense. I feel like I have a long way to go before I'm at the level I would have the expertise to be a leader”.

(Laura, pre‐placement interview)

Laura argued that whilst she would like to be a leader in her future career, she did not identify as one at the time, suggesting that becoming a leader was a long process which entailed gaining relevant expertise. Furthermore, she argued that, to become a leader, she would need to gain *“more confidence”* and *“more knowledge and ability to speak with people”*. Other students also displayed the same level of cautiousness. For example, Mary stated that *“she would like to gain more experience in different environments and departments, and gain more skills”* before becoming a leader, which is line with literature that views leadership as a form of apprenticeship (Kempster 2006).

Laura's views about becoming a leader remained mostly unchanged even after the placement, when she claimed that being in a leadership role was *“not that important to her at that moment”* as she had *“more learning to do”* before being *“worthy or suitable for leadership”*, demonstrating, therefore, a provisional leadership identity (Zaar et al. 2020). This result also supported that of Komives et al. (2006), who found that students in stage six (integration) of the LID model remained committed to a continual process of self‐development.

When asked whether she aspired to a leadership role, she commented:

“I guess it depends on what you mean by leadership role. If you're talking about a senior leadership role in a company, I don't aspire to that. I don't think I'd want that much responsibility. Maybe I've had a bad experience because I've been in a travel company during a pandemic. So, I feel that the responsibility they must bear during this time is huge”.

(Laura, post‐placement interview)

Whilst hardships can be seen as a leadership development accelerator (Longenecker and Insch 2019), Laura's experience suggested that witnessing an unprecedented challenge can also overwhelm individuals and hinder their professional development (DeRue and Wellman 2009). In fact, working for a travel company amid a global pandemic discouraged Laura from pursuing more senior leadership roles, and thus, had a somewhat negative impact on her leadership identity and aspirations.

## 6 Relationship Between Lived Experience and Leadership Identity Development

**FIGURE 5:**
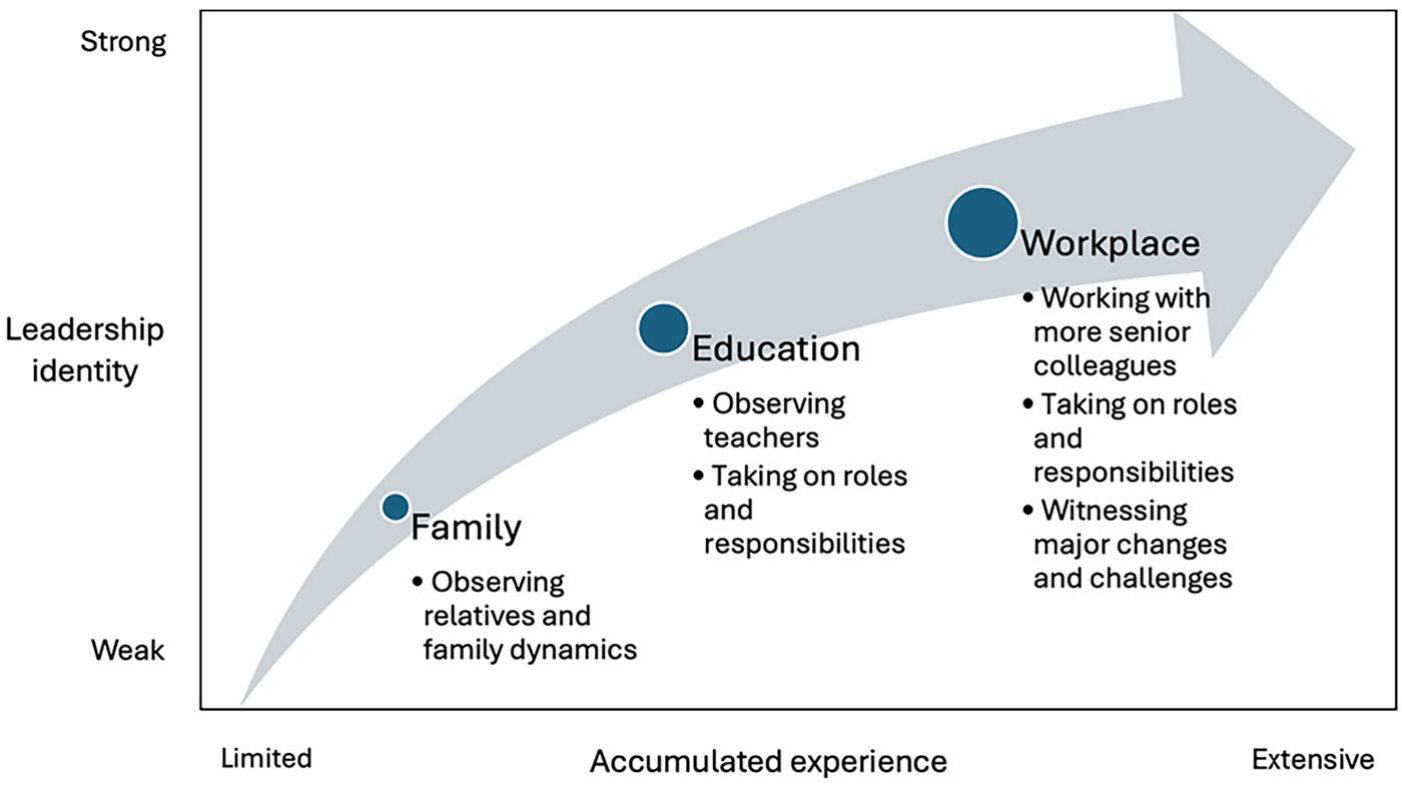
**Theoretical model**.

Drawing on Di Pede's (2024) model about how students learn to lead, Figure 5 illustrates the relationship between accumulated experience and leadership identity development.

This study has shown that through leadership experiences in different contexts over time, most students developed a stronger leadership identity. These experiences included observing both positive and negative role models as well as taking on roles and responsibilities in three main contexts, that is, family, education, and the workplace (Di Pede 2024). For example, most students identified teachers as leaders, and some of them, like Vivian, had taken on key leadership roles at university prior to their work placement. This in line with stage 1 and stage 2 of Komives et al. (2006) model, according to which students in their early stages of leadership identity development are able to recognize others as leaders as well as seek opportunities to lead themselves.

During the placement year, all students enhanced their understanding of leadership by working alongside more senior colleagues. More specifically, findings from this study suggested that the latter had considerable influence on the students’ leadership identity and aspirations, which confirms the importance of role models to leadership development (Di Pede 2024; Kempster and Parry 2014). This was particularly evident in Katie's narrative.

It is also worth noting how most students mentioned the Covid‐19 pandemic as a crucial experience, suggesting that witnessing major changes and challenges in the workplace may have a positive as well as negative impact on leadership identity development (DeRue and Wellman 2009; Di Pede 2024). In fact, whilst the majority regarded this event as a leadership development opportunity, some of them (e.g., Laura), were also intimidated by the amount of responsibility that leaders bore during these challenging times. Arguably, this affected their willingness as well as readiness to take on senior leadership roles in their future careers.

Nonetheless, this finding confirmed the importance of gaining experience over time to build confidence in one's own abilities to lead others. This is consistent with previous literature which argued that people who are more confident in their leadership skills, are more likely to actively pursue opportunities to lead, and thus, develop a strong leadership identity (Day and Sin 2011; Hammond et al. 2017; Zaar et al. 2020). Therefore, helping less experienced colleagues (novices) enhance their skills and self‐confidence through support and guidance is crucial to leadership development (Di Pede 2024).

## 7 Conclusion

This study offers several implications for leadership education and early‑career development, particularly for business schools, placement coordinators, and workplace supervisors seeking to support students’ leadership identity development through experiential learning.

Findings suggest that leadership identity development during placements is not an automatic outcome of work experience but depends on the quality and structure of students’ exposure to leadership in practice. Students developed stronger leadership identities when they were able to observe role models, participate in meaningful tasks, and make sense of these experiences over time. In practice, this may involve creating structured opportunities for leadership observation, such as shadowing supervisors, participation in group activities, such as meetings and projects, and reflection on experience.

The findings also highlight the importance of calibrated challenge in early‑career leadership development. Although responsibility and exposure to demanding tasks often enhanced students’ confidence and leadership identity, excessive challenge, especially in highly uncertain contexts, had the potential to overwhelm students and deter leadership aspirations. Therefore, workplace supervisors should progressively increase students’ responsibility over the placement year, ensuring that challenging tasks are accompanied by guidance, feedback, and support.

Leadership learning in this study often occurred in a tacit manner and became visible only through reflection. Without deliberate opportunities for sense‑making, students may struggle to recognise or integrate leadership learning into their developing identities. Educators and placement tutors should embed structured reflection mechanisms throughout the placement. Guided reflective journals, critical incident prompts, or facilitated debriefs during and after the placement can help students connect specific experiences to broader understandings of leadership and support identity integration across contexts.

An important implication concerns students’ use of “natural leader” language to describe themselves. Although such narratives were associated with strong leadership identities, they risk reinforcing the belief that leadership is innate rather than developed, potentially discouraging students with weaker identities. Thus, leadership educators should explicitly challenge these traditional conceptions of leadership, emphasizing leadership as learned, relational, and context‑dependent.

Finally, the study underscores the influence of role models, including female role models, on students’ leadership identity and aspirations. Observing leaders with whom students could identify appeared to strengthen confidence and shape future leadership intentions. Where possible, placement coordinators and employers should seek to increase students’ exposure to diverse leaders, including women in leadership roles, through supervision arrangements, mentoring relationships, or project work. This may be especially important in male‑dominated contexts and can support more inclusive leadership development trajectories.

Despite its contributions to both theory and practice, this study has some limitations. First, the small sample size and the fact that it involved business students from only one British university may not allow for generalizability of the results.

Second, the quality of the data may have been affected by reliance on memory as students were asked to recall leadership experiences before and after the placement. Thus, the results were subject to what the students could effectively remember during the interviews.

Third, the scope of this research was limited by the lack of information and data from the students’ line managers and colleagues. Future research could involve placement students as well as their supervisors. This would certainly add another perspective to the study of the impact of early‐career experiences on students’ leadership identity development by exploring how significant others view them in addition to how they see themselves.
